# The psychological mechanism of Self-objectification: the interaction between sociocultural pressures and the self-system

**DOI:** 10.3389/fpsyg.2025.1531222

**Published:** 2025-09-29

**Authors:** Zhennan Liu, Mei Fu, Jianmei Shi, Yinying Hu, Xiangping Gao

**Affiliations:** School of Psychology, Shanghai Normal University, Shanghai, China

**Keywords:** self-objectification, body surveillance, body dissatisfaction, self-concept clarity, sociocultural pressures

## Abstract

Self-objectification involves adopting an observer’s perspective on the body and prioritizing appearance over internal attributes, which is most common in women. We propose that self-objectification arises from the interplay between sociocultural pressures and the self-system rather than from sociocultural forces alone. In this account, self-concept clarity functions as a susceptibility factor that conditions the internalization of appearance norms; internalization and upward social comparison then increase body surveillance and appearance-focused negative affect (e.g., body shame, dissatisfaction). Over time, these experiences consolidate negative self-schemas and ruminative thinking, which help sustain and amplify self-objectification. Consequently, self-objectification is associated with reduced interoceptive awareness and compromised self-regulation, with downstream implications for cognition, mood, and health-related behaviors. We outline priorities for future research: (a) testing the moderating role of self-concept clarity across development, (b) clarifying how negative self-schemas and rumination maintain self-objectification over time, (c) distinguishing state versus trait/chronic forms and their effects on regulation, and (d) integrating mechanistic assays with intervention studies (e.g., mindfulness, self-compassion, cognitive reappraisal). Taken together, this framework highlights the intertwined influences of sociocultural environments and self-structure in the emergence and persistence of self-objectification.

## 1 Introduction

Many women often prioritize appearance over skills and personality traits, leading to self-objectification (Fredrickson and Roberts, 1997). Specifically, self-objectification refers to a self-schema that prioritizes appearance over other aspects of the self, leading individuals to assess their worth based on looks rather than abilities or inner qualities. This stance is enacted through body surveillance—the habitual, third-person monitoring of one’s appearance that highlights visible features over functional traits and often triggers appearance-focused rumination, a loop of self-critical, repetitive thinking about the body (Lindberg et al., 2006).

According to objectification theory, self-objectification can manifest as state self-objectification (SSO) or trait self-objectification (TSO) (Fredrickson and Roberts, 1997). SSO is a fleeting reaction to objectifying cues—such as a sexualized gaze, exposure to sexualized media, or even catching one’s reflection—whereas TSO reflects how often and how centrally such reactions recur in everyday life. Linder and Daniels (2018) identify two core processes: (1) internalizing the observer’s perspective on one’ s body and (2) treating the body as the primary index of the entire self. Calogero and Pina (2011) portray SSO as a ‘dual focus of attention,’ in which women view their bodies simultaneously from within and from an imagined outsider’ s vantage point, often accompanied by estrangement from the body. Although the label trait implies stability, empirical work shows that self-objectification fluctuates with developmental stage and cultural context (Tiggemann and Lynch, 2001) and is only weakly heritable (Moradi and Huang, 2008). To underscore this malleability, Winn and Cornelius (2020) prefer the term chronic self-objectification (CSO). Accumulating evidence also implicates repetitive negative thinking—especially ruminative self-focus—as a cognitive ‘glue’ that binds these experiences together and facilitates the shift from transient SSO episodes to longer-lasting CSO. Whether scholars foreground sociocultural pressures or individual dispositions, a single-factor lens has proved insufficient. We therefore propose that dynamic interactions among (a) sociocultural appearance pressures, (b) self-system (e.g., self-concept clarity, body-related schemas), and (c) maladaptive thinking modes such as rumination are key to understanding how self-objectification originates, consolidates, and endures.

Yet this theoretical blind spot is far from an academic nicety; it bears directly on a pervasive and high-risk public-health issue. It has shown that self-objectification is prevalent and is a high-risk factor for individual mental health and social stability development (Ward et al., 2023). For instance, approximately 66% of young women exhibit levels of self-objectification that surpass the average (Daniels et al., 2020). Among those with self-objectification, about 40% are at risk of developing eating disorders (Schaefer and Thompson, 2018), and about 20% are prone to experiencing depression (Jones and Griffiths, 2015). Self-objectification also increases socially aggressive behavior and decreases social vitality and creativity (Poon and Jiang, 2020). Researchers are investigating the psychological mechanisms of self-objectification, leading to various theoretical explanations. However, this diversity has sparked significant debate, complicating interventions aimed at addressing the issue.

In this review, we aim to elucidate the theoretical model and psychological mechanisms underlying the development, perpetuation, and adverse outcomes of self-objectification. Such insights can illuminate the detrimental cycle of self-objectification in individuals and offer theoretical foundations for its intervention. Specifically, we (a) synthesize evidence on how sociocultural appearance pressures initiate state self-objectification, (b) examine the moderating role of self-structure—particularly self-concept clarity and body-related schemas—in the transition from transient states to chronic tendencies, and (c) highlight the amplifying influence of ruminative thinking patterns that sustain and exacerbate body-focused concerns. By mapping these three interlocking processes, we provide an integrative framework that clarifies why existing single-factor accounts remain incomplete and where future empirical tests and clinical efforts should concentrate.

## 2 Theoretical models of self-objectification

Since being introduced as a pivotal framework in self-objectification, objectification theory has received extensive empirical backing (Fredrickson and Roberts, 1997). Simultaneously, it also encounters some challenges. For example, this theory is distinctly feminist, explaining how women self-objectify; however, evidence shows that men also engage in self-objectification (Oehlhof et al., 2009). There remains a lack of causal empirical evidence supporting the key stages of the self-objectification process, including internalization (Goldenberg and Roberts, 2011), body monitoring, and body dissatisfaction (Choma and Prusaczyk, 2018). In response, researchers have attempted to introduce alternative theoretical models to elucidate the psychological mechanisms behind self-objectification, such as Terror Management Theory (Goldenberg et al., 2009), Power Theory (Gruenfeld et al., 2008), System Justification Theory (Calogero, 2013), and Expectancy Theory (Teng et al., 2017).

### 2.1 The classic model: objectification theory

Objectification theory has several key points. First, it holds that self-objectification is a psychological phenomenon produced in the process of socialization, originating from Western societal culture, which has received empirical support (Gattino et al., 2023; Gattino et al., 2018). This culture is conceptualized as sexual objectification, separating the body from the individual, exaggerating the material function and value of the body, and equating the value of the individual with the value of the body, rather than the value as human beings (Baldissarri et al., 2022). For example, women in western society are often sexually objectified. They are constantly being watched and evaluated for the physical features of their bodies, rather than the abilities. These sexual objectifying stimuli (such as nudity, sexy postures, and appearance evaluation) can lead to self-objectification (Bernard et al., 2020). However, recent studies also suggest that self-objectification is not solely a Western phenomenon but rather a process influenced by a complex interplay of biological, psychological, and sociocultural factors across diverse cultural contexts (Gattino et al., 2023; Gattino et al., 2018). Additionally, evidence suggests that men engage in self-objectification as well (Karsay et al., 2018). This observation disrupts the commonly held view that self-objectification is mainly a trait associated with females.

Second, it posits that the internalization of sexual objectification shapes self-objectification. In essence, the pivotal factor in the emergence of self-objectification is the extent to which an individual internalizes these sexual objectification stimuli. Internalization entails not only agreeing with these values but also aligning one’s behaviors with them. This view has been supported by the finding that the higher the degree of internalization of sociocultural standards, the higher the degree of self-objectification (Hocking and Gondoli, 2023). However, it’s important to note that not everyone internalizes sexual objectification and then forms self-objectification.

Third, it holds that the core content of self-objectification lies in body surveillance and body dissatisfaction. Moreover, they are the mechanism by which self-objectification induces psychological problems and diseases. Cognitively, physical appearance occupies cognitive resources, that is, body surveillance (McKenney and Bigler, 2016). Emotionally, it is manifested as negative emotions pointing to physical appearance, such as body shame, body dissatisfaction, appearance anxiety and so on (Schaefer and Thompson, 2018). Evidence shows that the higher the degree of self-objectification, the higher the degree of body surveillance and body dissatisfaction (Teng et al., 2019) Studies support the significant roles of body surveillance and body dissatisfaction in the process of self-objectification (Lindner and Tantleff-Dunn, 2017; McKenney and Bigler, 2016). In addition, in a recent long-term follow-up study, self-objectification did not predict future body shame after accounting for past body shame (Kilpela et al., 2019). The findings suggest that while body objectification and body dissatisfaction are key psychological mechanisms of self-objectification, they may not be the sole factors involved.

Finally, Fredrickson and Roberts (1997) regard self-objectification as a high risk factor for mental health. It can lead to depression, anxiety, eating problems, substance abuse and other psychological problems and diseases. Evidence indicates a correlation between self-objectification and psychological problems such as depression and anxiety (Lindner and Tantleff-Dunn, 2017; Sun, 2021; Teng et al., 2019; Teng et al., 2021). However, recent studies have shown that self-objectification can not only induce psychological problems and clinical diseases, but also bring various negative consequences, including decreased cognitive performance (Kahalon et al., 2018) and increased negative emotions (such as shame, low self-esteem, anxiety)(Dvir et al., 2021), increased propensity for cosmetic surgery (Sun, 2021), and even eating disorders (Niu et al., 2020), addictive behaviors, and alcohol abuse behaviors (Baildon et al., 2021; Carretta and Szymanski, 2022). These negative consequences not only cover a wide range of cognitive, emotional, physical, and behavioral outcomes, but also occur more frequently in adolescence and early adulthood (Daniels et al., 2020).

### 2.2 Alternative theoretical models

Goldenberg et al. (2009) integrated Terror Management Theory with self-objectification, proposing that women engage in self-objectification as a means of regulating both the perception of their animalistic traits and the existential anxiety associated with mortality. This theory suggests that women inherently possess characteristics linked to nature, instinct, and physiological functions (e.g., reproduction and breastfeeding), which, in turn, heighten their awareness of mortality. For instance, the ability to give life through childbirth inherently underscores the reality of death. When mortality awareness is heightened, individuals tend to adopt rational strategies to emphasize their distinction from animals. In the case of self-objectification, societal and cultural norms—such as the expectation for women to wear makeup, high heels, and maintain a slim figure—serve as mechanisms to “tame” these perceived animalistic traits by regulating and monitoring the female body. Empirical studies have provided evidence supporting the relationship between self-objectification and mortality awareness. In one study, when participants were primed with death-related thoughts, they were more likely to perceive synthesized female faces as real, whereas this effect was not observed with male faces. More direct evidence comes from research indicating that when participants’ fear of death was activated, their levels of self-objectification increased (Morris et al., 2014). Additional evidence suggests that self-objectification functions as a means of suppressing animalistic traits. It has been reported that the more self-objectifying a woman is, the more likely she is to avoid bodily functions perceived as diminishing female attractiveness, such as menstruation and childbirth (Johnston-Robledo et al., 2007). According to Terror Management Theory, self-objectification is more prevalent among women. However, the theory fails to fully account for an important phenomenon: men also engage in self-objectification, experiencing body dissatisfaction and striving to attain an idealized height (Rahman and Navarro, 2023). This suggests that the underlying reason for self-objectification may not solely be the regulation of women’s animalistic traits and the management of existential fear.

Another theory integrated into the study of self-objectification is Power Theory (Gruenfeld et al., 2008). It holds that the key to the formation of self-objectification lies in power. That is, power, not sexuality, often plays a dominant role in sexual assault and harm. Those with high power objectify others more, while those with low power are objectified more. For those with low power, objectification represents a sense of oppression and powerlessness. Meanwhile, power affects self-construction, and people with low power are easy to form self-objectification. It is found that high power is accompanied by independent self-construction, which tends to be positive actions and strong goal execution (Guinote, 2017). Compared with high-power people, low-power people show more social conformity and less adherence to their own inner needs (Guinote, 2010). These findings suggest that that self-objectification is influenced by a complex interplay between sexual objectification and power dynamics. For people with low power, sexual objectification leads to self-objectification. In this framework, the sense of powerlessness may be the reason for the maintenance of the negative cycle of self-objectification. In the no-power posture situation, participants exhibited more problem eating behaviors, but this effect was reversed in the high-power posture (Allen et al., 2013). Compared with high-power sitting (upright sitting) and low-power sitting (slouching sitting), participants subjectively reported more negative emotions (Kozak et al., 2014). The sense of powerlessness stemming from low status prompts self-objectifiers to overlook their intrinsic needs. They constantly monitor themselves as if observed by others, leading to various negative psychological outcomes over time. While Power Theory’s explanation for self-objectification goes beyond gender and is not exclusive to women, it faces another challenge: individuals with high power tend to objectify both others and themselves (Inesi et al., 2014). Such results suggest that the root cause of self-objectification may not be solely linked to feelings of diminished power.

Calogero (2013) firstly applied System Justification Theory to self-objectification, which broadly addresses people’s inherent need to maintain the status quo (Jost and Banaji, 1994). Expanding on this framework, Calogero argued that women are often motivated to uphold a system that marginalizes them, for example, by resisting women’s rights movements and internalizing societal beauty standards as part of the prevailing system. For self-objectifiers, the need is not to change social injustice (e.g., the inequality between men and women), but to preserve it, even if it is not in their own interest (Calogero et al., 2014; Calogero et al., 2017). This is similar to fatalism in religion, where Christians are more receptive to systematic justice than atheists (Van der Toorn et al., 2017). Another instance involves women of color in India: the more they endorse the systemic belief that “white is beautiful,” the more closely they monitor their skin color and the more inclined they become to pursue skin bleaching (Choma and Prusaczyk, 2018). Although System Justification Theory can explain some phenomena of self-objectification, it has also been questioned. A recent study revealed that there was no significant correlation between women’s scores on self-objectification and their scores related to upholding systemic justice (i.e., perpetuating gender inequality). Additionally, there was no notable correlation between these scores and tendencies towards social activism (i.e., advocating for gender equality) (De Wilde et al., 2020). This result suggests that the explanation of self-objectification by maintenance System Justice Theory has its limitations and needs to be explained from a more comprehensive perspective.

Recently, Expectancy Theory proposes that the reason of self-objectification lies in the fact that individuals believe that a perfect appearance cannot bring benefits to themselves (Teng et al., 2017). Evidence shows that after adjusting for self-control and self-efficacy, women confident in their appearance have a brighter future outlook and anticipate smoother social progress (Wang et al., 2020). Drawing from Expectancy Theory, interventions were developed to shift women’s perceptions of appearance benefits, steering them to healthier goals. Such strategy notably diminished self-objectification, undermining its negative impact on women’s well-being (Teng et al., 2021). According to Expectancy Theory, profit-oriented people are more likely to objectify themselves. However, there is empirical evidence that women who highly value money do not always exhibit high scores of self-objectification (Teng et al., 2016). This result indicates that Expectancy Theory might not universally explain the psychological processes of self-objectification.

### 2.3 Summary of existing theoretical models

Although existing theoretical models of self-objectification have accumulated considerable empirical support, none currently offers a sufficiently integrative account that generalizes effectively across diverse gender and cultural contexts. A central limitation of these frameworks is their insufficient consideration of the dynamic, reciprocal interactions between sociocultural pressures and the structural and functional dimensions of the self-concept. Most prevailing theories either focus primarily on external sociocultural forces (e.g., sexual objectification in media, gender-based power dynamics) or emphasize individual-level factors (e.g., internalization of appearance ideals, expectancy-based motivations) without adequately capturing how these domains mutually shape and reinforce each other over time.

This conceptual division has resulted in fragmented and incomplete accounts of self-objectification. In particular, existing models struggle to explain: (a) the processes by which individuals—regardless of gender—actively internalize, negotiate, or resist sociocultural appearance norms; (b) individual differences in susceptibility to chronic self-objectification given identical sociocultural pressures; and (c) the developmental mechanisms through which transient states of self-objectification become entrenched as stable traits. Consequently, current frameworks fall short of elucidating the diverse trajectories and developmental pathways of self-objectification across various sociocultural, personal, and temporal contexts.

To address these theoretical gaps, we propose an integrative, developmentally oriented model emphasizing a reciprocal feedback loop involving sociocultural appearance pressures, characteristics of the self-system (e.g., self-identity, self-concept clarity), and maladaptive cognitive patterns (particularly appearance-focused rumination). In the sections that follow, we elaborate on the theoretical foundations, structural components, and developmental processes of this integrative framework, highlighting its explanatory potential for understanding and intervening in the complex phenomenon of self-objectification.

## 3 Developmental perspective: the self-system as a mediator of environmental pressures

Adolescence represents a sensitive window in which the self-system—comprising self-concept clarity, the content of self-concept, and identity commitment—consolidates and becomes the primary interface through which sociocultural appearance pressures are interpreted (Campbell et al., 1996; Harter, 1999). In this section, we argue that variation across these components helps explain why comparable objectifying environments propel some adolescents toward chronic self-objectification (CSO) whereas others remain resilient.

### 3.1 Self-identity

Self-identity denotes a coherent sense of who one is, integrating values, roles, group memberships, and self-attributes. It addresses the question “Who am I?” (Harter, 1999). Developmental shifts from concrete to abstract self-descriptions are detailed in Section 3.2 (Self-Concept). During adolescence, intensified social comparison and evaluative feedback foster greater attention to appearance norms; for some, these norms begin to structure identity content and heighten vulnerability to self-objectification.

Although work directly linking self-identity and self-objectification is limited, recent study identify critical connections (Cary et al., 2021), particularly around sexual self-concept and identity exploration. Cary and colleagues propose a theoretical pathway through which self-objectification negatively impacts self-concept, especially through sexual subjectivity, which in turn influences identity exploration. Their study highlights that self-objectification not only leads to lowered self-esteem but also significantly undermines sexual subjectivity—a core component of self-identity that encompasses positive sexual self-perceptions, entitlement to sexual pleasure, and sexual self-reflection (Horne and Zimmer-Gembeck, 2006). Specifically, women who engage in self-objectification are less likely to feel empowered in sexual situations (Horne and Zimmer-Gembeck, 2006), which suggests that self-objectification distorts their perceptions of sexual agency and reduces their ability to claim sexual pleasure, both of which are crucial for a healthy sexual self-identity. These patterns indicate that SO impairs not only body image but also broader identity development, including sexual self-concept formation and identity exploration.

### 3.2 Self-concept

Self-concept concerns the descriptive content of the self—abilities, traits, values, and appearance (Harter, 1999). In childhood, self-concept tends to be more concrete, with statements like “I can draw” or “I am good at sports.” During adolescence, self-concept becomes more abstract, with individuals describing themselves in terms of traits like “I am creative” or “I am thoughtful.” This shift is significantly influenced by social comparison, where adolescents evaluate themselves against their peers, and feedback from others (e.g., parents, teachers, friends). As adolescents mature, they start to internalize societal standards for appearance, which can profoundly shape their self-concept.

A key element of self-concept is self-concept clarity, which refers to the degree of consistency, stability, and clarity in an individual’s understanding of themselves (Campbell et al., 1996). Adolescents with high self-concept clarity can clearly define and articulate their attributes (e.g., “I am extroverted but not good at math”) and are less influenced by situational changes. In contrast, adolescents with low self-concept clarity often experience self-doubt and inconsistency in how they view themselves (e.g., “I am sometimes confident, but at other times I feel worthless”). Such fluctuations are especially pronounced in adolescence, when identity commitments are still forming and external pressures are salient; consequently, low SCC may magnify the impact of appearance-based feedback and increase susceptibility to SO.

Evidence has found a significant negative correlation between self-concept clarity and self-objectification, indicating that individuals with lower self-concept clarity tend to exhibit higher levels of self-objectification (Cui and Fang, 2022; Felig, 2024; Teng et al., 2016). Adolescents are especially vulnerable to sociocultural pressures that shape the self-concept, increasing the likelihood of internalizing appearance ideals. Internalization plays a critical mediating role in this process, as individuals who deeply internalize societal standards—such as the sexualization of women’s bodies—are more likely to base their self-worth on physical appearance rather than internal traits like abilities or personality (Fardouly et al., 2018; Vartanian et al., 2023). For example, research has shown that girls who internalize sexual objectification often view physical attractiveness as central to their self-worth, adjusting their preferences and behaviors to align with sexualized gender norms (Tobin et al., 2010).

Another case is that after internalizing the thinness ideal, participants subjectively reported placing more importance on physical appearance (Vartanian et al., 2023). This internalization is not simply an awareness (e.g., of what constitutes beauty in a particular culture) or recognition (e.g., agreement with cultural norms) of these standards, but rather a personal belief and pursuit. Consistent with this, higher SO is associated with beliefs that appearance matters more than ability (Vandenbosch et al., 2015). Self-objectifying women will constantly strive to make their appearance conform to sociocultural standards, devote time and resources to their appearance and regard it as an important goal for themselves, while lowering expectations for their academic performance (McKenney and Bigler, 2016).

Self-objectification is particularly prevalent in adolescence and early adulthood, a time when the self-system is still developing and has not yet fully matured. During this period, adolescents frequently compare themselves with peers and media figures when evaluating self-worth; social media platforms amplify exposure to idealized “perfect bodies,” encouraging upward comparisons to influencers and celebrities (Vogel et al., 2014). Exposure to such ideals can foster the belief that being thin or beautiful is necessary for self-worth. Social comparison theory suggests that this upward comparison can lead adolescents to incorporate these ideals into their self-concept, which further entrenches self-objectification.

Moreover, adolescents’ self-concept is particularly malleable, making it easy for them to adopt societal ideals (e.g., the “perfect body” praised on social media) as normative rather than recognizing them as socially constructed standards (Bell et al., 2018). Studies have demonstrated that adolescents who spend over 3 h per day on social media perceive a 27% increase in the gap between their actual body type and their ideal body (Perloff, 2014). A strong need to belong in adolescence can further intensify these processes, as youths may prioritize conformity to group norms over personal values or needs, reinforcing self-objectification (Daniels et al., 2020).

## 4 The important role of rumination in the formation of self-objectification and the development of symptoms

Rumination is a cognitive pattern in which individuals repetitively, passively, and compulsively focus on their negative emotions (such as sadness and anger) and their triggers, consequences, and related thoughts. The essence of rumination is a pathological cognitive pattern that “loops inward,” and its core features include: self-directedness, with thoughts focusing on questions like “Why do I feel this pain?” or “Where is the problem?” related to frustrating events; passivity, where the thinking process remains at the “repetitive thinking” level without the inclination to take problem-solving actions; emotional intensification, which further exacerbates negative emotions such as depression and anxiety (Kirkegaard Thomsen, 2006), through repetitive processing of negative information; and temporal duration, where the thinking process can last for hours, days, or even months (Nolen-Hoeksema et al., 2008; Watkins and Roberts, 2020).

Prior research indicates that women who exhibit increased rumination (closed cognition, characterized by a propensity to dwell on negative thoughts) following encounters with sexism tend to experience heightened levels of body monitoring and body dissatisfaction (Calogero and Jost, 2011). The higher an individual’s level of self-objectification, the more serious his rumination (repeated thinking about his own body), and rumination mediated the negative impact of self-objectification on subjective well-being (Jarrar, 2017). These results support the correlation between self-objectification and self-rumination. Specifically, the higher the self-objectification, the heavier the rumination thinking.

Self-rumination might strengthen the cognitive bias of self-objectification. Evidence shows that self-objectification can reduce the math test scores of the participants (Gervais et al., 2011; Kahalon et al., 2018; Pacilli et al., 2016). Additionally, rumination exacerbates the clinical symptoms of self-objectification. A large amount of empirical evidence shows that that self-objectification is predictive of individual symptoms of depression (Milan and Perez, 2021; Prusaczyk and Choma, 2018; Stanton et al., 2022), anxiety (Calogero et al., 2014; Fitzsimmons-Craft and Bardone-Cone, 2012; Hanna et al., 2017), and other negative emotions (Carrotte and Anderson, 2019; Teng et al., 2019).

## 5 An integrative model of the interaction between sociocultural pressures, self-concept degradation, and rumination

**Figure 1:**
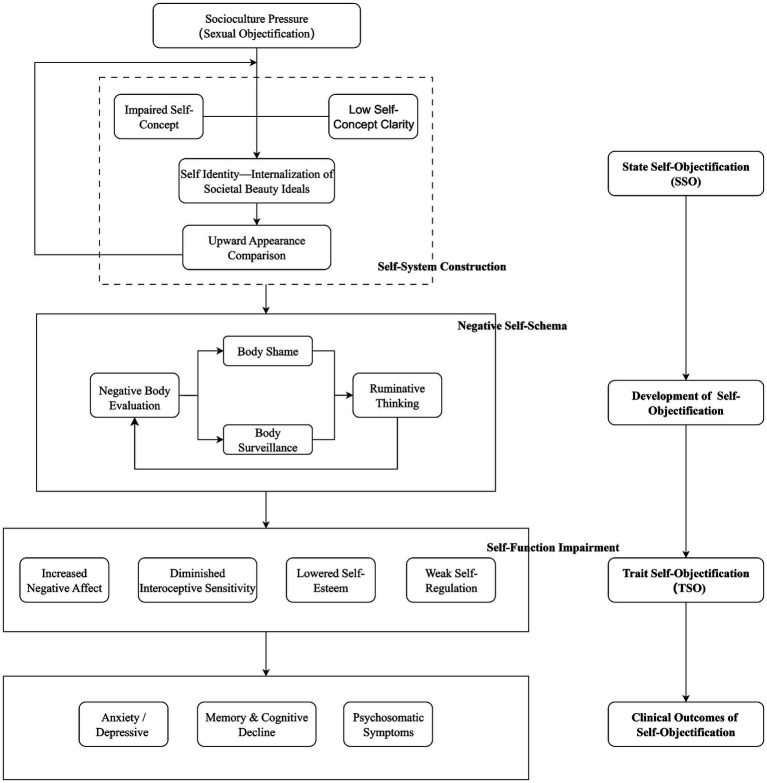
An integrative model of self-objectification. The solid line denotes areas supported by existing evidence, while the dotted line highlights aspects that necessitate further investigation and clarification.

In this review, we propose an integrative model that illustrates how sociocultural pressures, self-concept degradation, and rumination interact to contribute to the development and maintenance of self-objectification and related psychological symptoms. We argue that these factors do not act in isolation but rather interact dynamically, exacerbating the negative effects of each other. (Figure 1).

Specifically, self-objectification is strongly shaped by sociocultural pressures—especially sexual objectification in media and interpersonal contexts. When individuals have an ambiguous self-concept, marked by low self-concept clarity, they are more likely to internalize societal beauty standards. This internalization process leads to an adoption of these standards as part of their identity. As a result, individuals engage in upward appearance comparisons, exacerbating the impact on their self-concept. This continual comparison with sociocultural beauty ideals contributes significantly to self-system construction, particularly for adolescents whose self-systems are not fully matured. In this context, the internalized beauty ideals reinforce self-objectification. Adolescents, in particular, are more susceptible to this process due to the inherent instability and malleability of their self-concept during development.

Once self-objectification takes root, it triggers negative self-evaluation, often focused on appearance, which leads to body surveillance. This cycle of body monitoring frequently results in negative emotions, such as body shame and appearance anxiety. These emotions further entrench the negative self-schema formed through self-objectification.

As the process continues, rumination intensifies, reinforcing the negative self-schema. This, in turn, exacerbates the ongoing development of self-objectification. When self-objectification becomes stable and consistent, it solidifies into trait self-objectification (TSO). Individuals with TSO experience increased negative emotions, such as depression and anxiety, along with lowered self-esteem, diminished interoceptive awareness, and weakened self-regulation abilities. These impairments contribute to a range of clinical symptoms, including cognitive decline, somatization, and psychosomatic symptoms.

The cycle outlined in this model illustrates how sociocultural pressures, through self-concept degradation and rumination, can progressively contribute to the development and deepening of self-objectification, culminating in significant emotional and psychological consequences. In the next sections, we will elaborate on the key components and underlying mechanisms driving this process.

### 5.1 Sociocultural pressures and individual differences in self-objectification development

As previously discussed, we also contend that the origin of self-objectification lies in sociocultural pressures, particularly in relation to cultural norms surrounding physical appearance. This notion has received substantial theoretical and empirical support (Ata et al., 2015; Karsay et al., 2018; Tiggemann and Andrew, 2012; Vandenbosch et al., 2015). For example, several theoretical frameworks have been proposed to explain these sociocultural influences, including Stice’s Dual Pathway Model (Stice and Shaw, 1994), the Tripartite Influence Model (Thompson et al., 1999), and Objectification Theory (Fredrickson and Roberts, 1997). A commonality among these models is that they all position sociocultural influences as foundational constructs. In particular, these influences often take the form of sexual objectification, which reduces women to mere bodies or a collection of body parts (Bernard et al., 2020). Women are routinely subjected to sexual objectification in their everyday lives through television programs, music videos, films, and social media platforms such as Instagram and Facebook, as well as in daily interpersonal interactions. This exposure includes sexualized imagery, such as nudity, provocative poses, and appearance-based evaluations. For example, an analysis of 662 television programs found that 96% of female characters were depicted in revealing attire or partially nude, compared to 68% of male characters (Flynn et al., 2015). Similarly, a multinational study revealed that women aged 18 to 46 experienced an average of 2.7 instances of interpersonal sexual objectification over a five-day period (Koval et al., 2019). Individuals who internalize these sociocultural influences are more likely to develop self-objectification (Morry and Staska, 2001; Vandenbosch and Eggermont, 2012).

However, the impact of these sociocultural influences is not uniform across individuals. For example, Fredrickson et al. (1998) induced feelings of sexual objectification by having participants try on swimsuits and found that women with lower self-objectification traits reported less body shame during objectification than those with higher self-objectification traits. Similarly, Tiggemann (2011) reviewed experiments reporting that exposure to sexualized models increased objectification of self and others, with stronger effects among women higher in TSO. However, this effect was most pronounced among women with higher self-objectification traits. These findings suggest that women with lower self-objectification may be less vulnerable to the negative effects of sexual objectification, though the underlying cognitive and neural mechanisms that contribute to this resilience remain unclear. Additionally, recent research (Liu et al., 2025) has shown that even a simple cognitive reappraisal of sexually objectified models can reduce individuals’ attraction toward them, providing further insight into how individuals can mitigate the effects of sexual objectification through adaptive cognitive strategies.

Thus, while sociocultural pressures are powerful in shaping self-objectification, individual differences in self-objectification traits—such as cognitive reappraisal ability and self-regulation—play a crucial role in moderating these effects. These differences highlight the need to understand the complex interaction between societal influences and individual self-systems.

### 5.2 Self-system development impairments in transforming objectified societal values into self-concept

Research indicates that self-concept clarity influences the relationship between money worship and self-objectification. Specifically, among women with a high self-concept clarity, their levels of self-objectification remain lower even when they exhibit tendencies towards money worship (Teng et al., 2016). In addition, self-objectification often emerges during the teenage years and continues into the initial stages of adulthood, marked by the ongoing evolution of self-identity and the intrinsic volatility of one’s self-concept (Daniels et al., 2020). More direct evidence is that the clarity of one’s self-concept negatively correlates with the internalization of sexual objectification (Vartanian and Hayward, 2018; Vartanian et al., 2023). In our recent study, we found that self-concept clarity moderated the relationship between social networking site use and appearance anxiety—a key correlate of self-objectification (Hu et al., 2025). Specifically, both overall and active social networking site use predicted higher levels of appearance anxiety only among individuals with low self-concept clarity. Passive use predicted appearance anxiety regardless of clarity level, but the effect was stronger for those with low clarity. These findings suggest that low self-concept clarity amplifies individuals’ sensitivity to objectifying environments and their messages, rendering them more vulnerable to appearance-based anxiety and self-objectification.

Crucially, self-concept clarity is not merely an outcome of identity development—it actively shapes it. Schwartz et al. (2011) provide longitudinal evidence that self-concept clarity and identity commitments form a reciprocal, day-to-day feedback loop. In a diary study of 580 Dutch adolescents, higher self-concept clarity on 1 day predicted stronger identity commitments the next, and vice-versa; moreover, greater daily instability in either construct forecasted higher anxiety and depression 1 year later. Theoretical synthesis by Schwartz et al. (2018) further frames identity processes (exploration, commitment, reconsideration) as the active agent that “authors” a clear self-concept; stalled or repetitive exploration leaves self-concept clarity diffuse, rendering the self vulnerable to external objectifying messages.

Moreover, other factors that shape an individual’s level of self-objectification—such as self-compassion (Coutts et al., 2023; Miyagawa, 2024; Schwartz et al., 2018) and personality traits(Diehl and Hay, 2011)—exert their influence largely by altering self-concept clarity and identity status.

Together, these findings indicate that when self-concept clarity is low—and when protective resources such as self-compassion or adaptive personality profiles are lacking—objectifying cues are more easily internalized. This, in turn, sets the stage for the negative self-schemas and ruminative thought patterns examined in the next section.

### 5.3 The pivotal impact of negative self-schema and rumination in self-objectification

Furthermore, we propose that individuals who engage in self-objectification often develop negative self-schemas. Self-schemas refer to individuals’ conceptualizations of who they are, encompassing beliefs about their abilities, values, and typical patterns of behavior—what the professional literature refers to as schemas. Schemas are defined as “cognitive generalizations about the self, derived from past experience,” that “organize experience and action,” with content “reflected in implicit rules, attitudes, beliefs, and assumptions that determine the substance of thought, emotion, and behavior” (Cash and Labarge, 1996). Individuals assign varying degrees of importance to different aspects of the self, which in turn shapes how information is processed.

Once a negative body-focused self-schema takes hold, it quickly becomes the engine of rumination—a repetitive, passive interrogation of “What is wrong with my body?” or “Why am I not attractive enough?” Each perceived flaw re-activates the schema and prolongs rumination, locking the individual into a self-referential loop (Nolen-Hoeksema, 1991; Nolen-Hoeksema et al., 2008). The process hijacks attentional resources, crowding working memory with appearance-related thoughts and impairing higher-order cognition (van Vugt and van der Velde, 2018; Whitmer and Banich, 2007); it simultaneously amplifies affect, as the mind’s continual replay of negative body imagery intensifies shame and anxiety, further eroding self-esteem (Adams et al., 2017; Hanna et al., 2017; Watt and Konnert, 2020). Over time, this schema–rumination complex migrates into broader psychopathology, forecasting depressive mood (Berman et al., 2011; Olatunji et al., 2013), disordered eating (Delaney et al., 2015; Palmieri et al., 2021), and social withdrawal (Li et al., 2024).

### 5.4 Impaired self-functioning and environmental adaptation

Finally, we argue that self-objectifiers often develop negative self-schemas and engage in ruminative thinking, both of which may contribute to impaired self-functioning. This declines primarily manifests in two ways: firstly, a diminished awareness of one’s own body and inner needs, termed as interoceptive awareness. Secondly, a weakened self-regulation capability, affecting the individual’s ability to adjust cognition, emotions, and behavior in line with shifting environmental demands.

Evidence indicates that self-objectification is closely related to the decline of interoception (Myers and Crowther, 2007; Zhang and Yang, 2024). It has been reported that the higher the level of self-objectification, the lower the accuracy of an individual’s perception of their own heartbeat, suggesting that self-objectification is associated with lower interoception (Ainley and Tsakiris, 2013). In another study, when the researchers asked the participants to wear a small amount of clothing in a cold outdoor, the higher the level of self-objectification, the lower the subjective reported cold score, which indicates that self-objectification can weaken the perception of the individual’s body (Felig et al., 2022). In a recent neuroimaging study, elevated levels of self-objectification were found to correlate with reduced spontaneous activity in the right inferior frontal gyrus, thereby mediating the relationship between self-objectification and interoception (Du et al., 2023).

Self-objectification is also closely associated with a decline in self-regulation. Quinn et al. (2006) used the classic Stroop task to investigate the impact of self-objectification on self-regulation. The results showed that after activating self-objectification, the task performance of the participants became worse. A series of subsequent empirical studies replicated the above findings (Baker and Blanchard, 2017; Ching and Wu, 2023; Kahalon et al., 2018).

## 6 Discussion

The self is a distinctive psychological construct with a dual individual–social nature. Its development is enriched by the dynamic exchange between person and environment—an interplay of subject and object (Decety and Chaminade, 2003). Although this exchange is often balanced, imbalances can occur when external forces outweigh personal agency. Self-objectification exemplifies this dilemma: sociocultural pressures overshadow individual autonomy and, over time, can distort adaptation to one’s environment. At the core lie disruptions within the self-system—especially imbalances in self-concept and broader self-functioning—manifesting across cognition, affect, behavior, and other psychological processes. Numerous theories illuminate parts of this process, yet each provides only a partial account. We therefore advance a sociocultural adaptation model of self-objectification that centers reciprocal interactions between sociocultural influences and the self-system in the formation and development of self objectification.

### 6.1 Clinical significance of self-objectification

In our model, self objectification can culminate in clinically relevant symptoms across multiple domains. Emotionally, SO has been linked to depression and anxiety (Jones and Griffiths, 2015; Lamp et al., 2019; Peat and Muehlenkamp, 2011) and anxiety (Adams et al., 2017; Dimas et al., 2021), Cognitively and behaviorally, SO relates to poorer cognitive performance and problematic behaviors such as internet addiction Physically, self objectification is associated with eating disorders (Calogero et al., 2005; Hu et al., 2024; Schaefer and Thompson, 2018; Winn and Cornelius, 2020) and alcohol abuse (Carretta and Szymanski, 2022).

Once these clinical symptoms manifest, they are often resistant to intervention through general cognitive therapies. Therefore, the early recognition and intervention of self-objectification is crucial in preventing these disorders from developing further. Recent research has focused on interventions targeting self-objectification, such as through self-compassion (Wang et al., 2022) and mindfulness (Chen et al., 2025). While these approaches attempt to address the issue from the perspective of self-regulation, they lack a coherent theoretical framework that integrates the development of self-objectification with broader self-concept dynamics. This paper offers a more systematic approach by linking the development of self-objectification with individual self-development, providing a clearer theoretical foundation for future interventions. The shared developmental pathways between self-objectification and self-concept evolution, as explored in this model, can guide the design of targeted, evidence-based intervention strategies.

### 6.2 Self-development perspective on the necessity of studying self-objectification

As discussed earlier, self-objectification is a developmental process that is heavily influenced by both sociocultural pressures and individual psychological mechanisms. Research has consistently shown that although self-objectification can emerge at early stages, it is most prominent during adolescence, particularly between the ages of 11 and 16. This critical period is marked by the developmental phenomenon of identity confusion and increased social needs, which is when adolescents are most vulnerable to self-objectification (Daniels et al., 2020). The need for belonging, combined with the quest for social validation, increases adolescents’ sensitivity to societal beauty standards, which significantly shapes their self-concept.

Adolescence is a period when the self-concept is still forming and can be significantly influenced by external sociocultural messages. During this time, individuals are trying to establish their identities, often going through periods of exploration and role confusion (Erikson, 1959). For adolescents, the fluidity of self-concept, combined with high social pressures and exposure to objectifying media, makes them particularly susceptible to internalizing societal standards of physical appearance. Thus, the risk of self-objectification is heightened during this stage of self-development.

Research on self-development, particularly on self-concept clarity and identity formation, highlights the role of self-concept in shaping individuals’ experiences and their vulnerability to societal influences. Adolescents with a low level of self-concept clarity are more likely to internalize societal ideals of beauty and appearance, which in turn leads to self-objectification. This model suggests that self-objectification is not merely a passive consequence of societal pressures but an active process of self-concept development.

Therefore, studying self-objectification from the perspective of self-development is essential for understanding its underlying mechanisms and long-term impacts. Recognizing the factors that contribute to self-objectification during adolescence—such as identity confusion, social comparison, and the internalization of external beauty standards—will allow for more targeted interventions aimed at mitigating these effects. Furthermore, the relationship between self-concept development and self-objectification highlights the importance of early intervention during adolescence to prevent the onset of negative outcomes, such as body image issues, depression, and eating disorders.

### 6.3 Future research directions: empirical testing and refinement of the proposed model

In this section, we outline future research directions aimed at further empirically validating and refining the proposed model of self-objectification. Our model conceptualizes self-objectification as a dynamic process shaped by the interaction of sociocultural pressures and individual psychological mechanisms, including self-concept clarity, negative self-schemas, self-rumination, and impaired self-regulation. While several components of this model, particularly the role of sociocultural pressures, are well-supported by existing theoretical frameworks and empirical evidence, others remain underexplored. Specifically, the moderating role of self-concept clarity, the mechanisms involving negative self-schemas and repetitive thinking, and the broader implications for self-regulation require more rigorous investigation.

#### 6.3.1 Examining the moderating role of self-concept clarity

One key area for future research is examining how self-concept clarity moderates the relationship between external sociocultural pressures and self-objectification. Our model suggests that individuals with low self-concept clarity are more likely to internalize societal standards of appearance and engage in self-objectification. Research has already indicated that self-concept clarity influences how individuals process and internalize external pressures, with those exhibiting lower self-concept clarity more susceptible to these pressures (Teng et al., 2016). Future studies could track changes in self-concept clarity and the internalization of appearance ideals across different stages of development, particularly focusing on adolescents and young adults who are in the process of forming their identities. This could involve longitudinal studies to examine how fluctuations in self-concept clarity correspond to changes in self-objectification and its associated outcomes, such as body shame, appearance anxiety, and reduced self-esteem (Vartanian and Hayward, 2018; Vartanian et al., 2023). Researchers could also explore how self-concept clarity interacts with other individual traits, such as self-esteem, materialism, and social conformity, to influence susceptibility to self-objectification.

#### 6.3.2 Testing the role of negative self-schemas and rumination

Another critical avenue for future research involves understanding the role of negative self-schemas and rumination in the self-objectification process. As proposed in our model, negative self-schemas—particularly those related to body image—may serve as a catalyst for self-objectification by reinforcing negative self-evaluations and creating cognitive biases. These biases, particularly in attention and memory, may make individuals more likely to focus on appearance-related stimuli and engage in repetitive negative thinking (Wittenborn et al., 2016). Research has already shown that self-objectification is associated with negative body image and low self-esteem (Adams et al., 2017). However, it remains unclear how rumination exacerbates these effects over time.

Future studies could investigate how negative self-schemas develop and evolve over time, with particular attention to how they interact with rumination. This could involve experimental designs that prime individuals with objectifying stimuli and measure rumination and its effects on attention, memory, and emotional processing. Additionally, neuroimaging techniques such as fMRI and EEG could be used to examine the neural mechanisms underlying the interplay between negative self-schemas, rumination, and cognitive bias. Understanding these mechanisms will provide insight into the cognitive-affective processes that perpetuate self-objectification and identify potential targets for intervention.

#### 6.3.3 Investigating the impact of self-objectification on self-regulation

Finally, future research should examine how self-objectification affects self-regulation across different domains, such as emotional regulation, cognitive functioning, and behavioral control. Our model proposes that self-objectification undermines self-regulation, particularly in how individuals manage their emotions, attention, and behavior in response to social and environmental demands. Empirical evidence suggests that self-objectification can impair cognitive performance (Gapinski et al., 2003) and emotional regulation (Kahalon et al., 2018). However, the specific mechanisms through which self-objectification affects self-regulation need further exploration.

Future studies could investigate how different types of self-objectification—trait versus state—affect self-regulation. Self-objectification is often conceptualized as either a stable trait or a transient state (Vandenbosch et al., 2015). Understanding how these forms of self-objectification interact with self-regulation processes is crucial for identifying when and how interventions should be applied. For example, state self-objectification may primarily affect emotional regulation in the moment, whereas trait self-objectification may impair long-term self-regulatory capacity. Interventions aimed at improving self-regulation in individuals with high levels of self-objectification could include strategies such as mindfulness, cognitive reappraisal (Liu et al., 2025), and emotion regulation training.

#### 6.3.4 Integrating interventions and neurobiological research

As noted in our model, self-objectification is a dynamic process influenced by both sociocultural pressures and individual psychological mechanisms. Future research should examine how interventions, such as mindfulness-based programs, self-compassion training, and cognitive-behavioral therapies, can alter the trajectory of self-objectification and its associated negative outcomes. Additionally, neurostimulation techniques, such as transcranial magnetic stimulation and transcranial direct current stimulation, could be explored to target the neural pathways associated with self-referential processing and emotion regulation, offering potential for neurocognitive interventions to disrupt maladaptive cognitive-affective loops that perpetuate self-objectification.

By integrating empirical research on cognitive and emotional processes, neurobiological mechanisms, and interventions, future studies can help refine the proposed model and advance our understanding of self-objectification’s impact on individuals. This will provide valuable insights into how interventions can be tailored to address the specific cognitive, emotional, and behavioral challenges faced by self-objectified individuals, offering pathways to more effective prevention and treatment strategies.

## 7 Conclusion

In conclusion, this article advances an integrative sociocultural-adaptation model of self-objectification that specifies how sociocultural appearance pressures interact with the self-system—particularly self-concept clarity, negative self-schemas, rumination, and self-regulation—to shape the onset and maintenance of self-objectification across development. The framework clarifies why vulnerability and trajectories differ across individuals and links self-objectification to emotional, cognitive, and health-relevant outcomes via interoceptive and regulatory impairments. It also yields testable predictions and points to mechanism-targeted, developmentally timed interventions (e.g., strengthening self-concept clarity, reducing rumination, and enhancing interoceptive awareness).
